# Neutrophil Gelatinase-Associated Lipocalin (NGAL) in Patients with ST-Elevation Myocardial Infarction and Its Association with Acute Kidney Injury and Mortality

**DOI:** 10.3390/jcm12113681

**Published:** 2023-05-26

**Authors:** Mathias Alexander Højagergaard, Rasmus Paulin Beske, Christian Hassager, Lene Holmvang, Lisette Okkels Jensen, Yacov Shacham, Martin Abild Stengaard Meyer, Jacob Eifer Moeller, Ole Kristian Lerche Helgestad, Peter Dall Mark, Rasmus Møgelvang, Martin Frydland

**Affiliations:** 1 Department of Cardiology, The Heart Centre, Copenhagen University Hospital, Rigshospitalet, 2100 Copenhagen, Denmark; mathias.alexander.hoejagergaard@regionh.dk (M.A.H.);; 2 Department of Cardiology, Odense University Hospital, 5230 Odense, Denmark; 3 Department of Cardiology, Tel Aviv Sourasky Medical Center and Sackler Faculty of Medicine, Tel Aviv University, Tel Aviv 6997801, Israel; 4 Department of Clinical Biochemistry, Copenhagen University Hospital, Rigshospitalet, 2100 Copenhagen, Denmark; 5 Department of Clinical Physiology and Nuclear Medicine, Copenhagen University Hospital, Bispebjerg, 2400 Copenhagen, Denmark; 6 Centre for Cardiac, Vascular, Pulmonary and Infectious Diseases, Rigshospitalet, 2100 Copenhagen, Denmark

**Keywords:** Neutrophil gelatinase-associated lipocalin, NGAL, ST-elevation myocardial infarction, STEMI, acute myocardial infarction, acute kidney injury, AKI, mortality, biomarker, plasma

## Abstract

Neutrophil gelatinase-associated lipocalin (NGAL) is an inflammatory biomarker related to acute kidney injury (AKI). Including 1892 consecutive patients with ST-elevation myocardial infarction (STEMI), in which NGAL was measured in 1624 (86%) on admission and in a consecutive subgroup at 6–12 h (n = 163) and 12–24 h (n = 222) after admission, this study aimed to evaluate the prognostic value of NGAL in predicting AKI and mortality. Patients were stratified based on whether their admission NGAL plasma concentration was greater than or equal to/less than the median. The primary endpoint was a composite of the first occurrence of AKI or all-cause death within 30 days. AKI was classified by the maximal plasma creatinine increase from baseline during index admission as KDIGO1 (<200% increase) or KDIGO23 (≥200% increase) according to the Kidney Disease Improving Global Outcomes (KDIGO) system. Admission NGAL > the median was independently associated with a higher risk of severe AKI (KDIGO2-3) and 30-day all-cause mortality when adjusted for age, admission systolic blood pressure and high-sensitivity C-reactive protein, left-ventricular ejection fraction, known kidney dysfunction, and cardiogenic shock with an odds ratio (95% confidence interval) of 2.26 (1.18–4.51), *p* = 0.014. Finally, we observed increasing predictive values in a subgroup during the first day of hospitalization suggesting that assessment of NGAL should be delayed for optimal prognostic purposes.

## 1. Introduction

Ischemic heart disease is the most frequent cause of death globally [1]. Of surviving patients with acute myocardial infarction, up to 26% develop acute kidney injury (AKI) during hospitalization [2]. AKI is associated with increased mortality in patients with ST-elevation myocardial infarction (STEMI) [3].

NGAL is a biomarker secreted from neutrophils of the innate immune system in response to inflammation and renal ischemia [4,5]. High blood concentrations of NGAL have previously been associated with AKI [3,6] and all-cause mortality in small cohorts of STEMI patients [7,8,9,10].

Due to the early accumulation and detectability of circulating NGAL compared with creatinine [11,12], NGAL is a promising biomarker in early prediction of AKI. While creatinine is the all-time gold standard for detecting kidney injury in real time, NGAL may predict future AKI, allowing us to limit kidney injury and improve the general prognosis of STEMI patients.

In this study, we assessed the association between NGAL plasma concentration on admission and the development of AKI and all-cause death within 30 days in STEMI patients. Additionally, the study evaluated the temporal changes of NGAL plasma concentration after admission and the prognostic value hereof.

## 2. Materials and Methods

**Figure 1:**
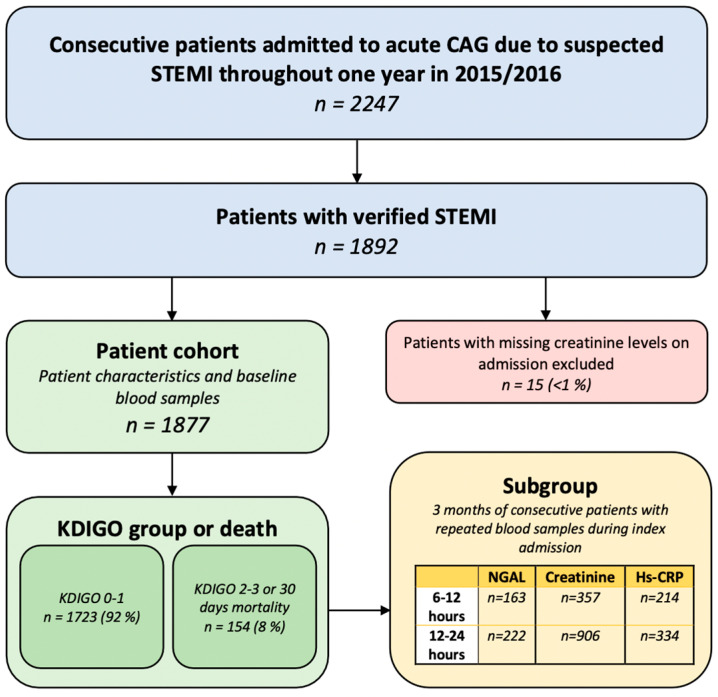
Flow diagram depicting inclusion of patients in the cohort and subgroup. Patients were included in the study if they had verified STEMI and available plasma creatinine levels on admission. Outcomes of patients were based on the level of AKI according to the KDIGO classification system and/or death within 30 days. In a subgroup of included STEMI patients, biomarkers were measured again at 6–12 and 12–24 h after admission. CAG = coronary angiography. STEMI = ST-elevation myocardial infarction. KDIGO = Kidney Disease Improving Global Outcomes (classification system). NGAL = Neutrophil gelatinase-associated lipocalin. Hs-CRP = High-sensitivity C-reactive protein.

This study population (Figure 1) has previously been described in detail [13]. Briefly, this prospective, observational study aimed to include a consecutive cohort of patients with STEMI from two tertiary heart centers in Denmark (Copenhagen University Hospital, Rigshospitalet, and Odense University Hospital) over one year in 2015 and 2016.

### 2.1. Study Population

On arrival in the catheterization laboratory, patient characteristics, hemodynamic parameters, and time from symptom debut to acute coronary angiography (CAG) were registered. During a 3-month period from January to March 2016, repeated plasma samples and biochemical measurements were obtained in a consecutive subgroup of patients with STEMI admitted at Rigshospitalet (Figure 1).

#### Kidney Disease Improving Global Outcomes (KDIGO) Criteria and 30-Day All-Cause Mortality

The definition and level of AKI were based on the serum creatinine criteria of the KDIGO classification system [14]. In this study, patients were stratified according to the maximal increase in plasma creatinine from baseline during index admission:

1. No AKI (KDIGO0): No increase of plasma creatinine (pCr).
2. KDIGO1: <200% pCr increase.
3. KDIGO2-3: ≥200% pCr increase.

Patients were stratified into two composite groups: KDIGO0-1 and no death versus KDIGO2-3 or death from any cause within 30 days (Figure 1).

### 2.2. Blood Sampling and Biomarker Analysis

On arrival in the catheterization laboratory, arterial blood samples were taken immediately after sheath insertion before the CAG. Blood samples were collected in lithium-heparin tubes, centrifuged at 2000 G, and plasma was stored at –80 °C.

Time points for temporal biomarker measurements in the subgroup were 0 h (baseline), 6–12 h, and 12–24 h after admission.

NGAL plasma concentrations were measured with an NGAL Test^TM^ Reagent Kit (Roche Cobas 6000 C501/C502; Rotkreuz, Switzerland) according to the manufacturer’s instructions [15]; inter-assay coefficients of variability (CVs) were 11.8% (88 ng/mL), 5.4% (239 ng/mL), and 3.7% (2662 ng/mL) and intra-assay CVs were 7.5% (62 ng/mL), 2.1% (235 ng/mL) and 1.5% (2772 ng/mL). High-sensitivity C-reactive protein (hs-CRP) serum concentrations were measured using a CRP-HS assay (Cobas, by Roche, Basel, Switzerland) according to the manufacturer’s instructions; inter-assay CVs were 6.8% (3.06 mg/L) and 3.8% (1.00 mg/L), and intra-assay CVs were 4.0% (3.44 mg/L) and 0.9% (9.14 mg/L). Plasma creatinine concentrations were assayed by routine laboratory methods.

### 2.3. Statistical Analysis

Categorical variables are presented as numbers (n) and percentages (%). Pearson’s Chi-squared test or Fisher’s exact test were used accordingly to assess differences between categorical variables. Continuous variables are presented as the mean with standard deviation (SD) or the median with 25th and 75th percentile (25th; 75th). Differences between continuous variables were tested using the Wilcoxon rank test or Kruskal–Wallis rank sum test for >2 groups.

Patients were stratified into > versus ≤ the median of admission NGAL plasma concentration. The cumulation of KDIGO2-3 and 30-day all-cause mortality was used as the composite endpoint.

The association between admission NGAL plasma concentration and composite endpoint was assessed using logistic regression in a univariable and multivariable model (adjusted for age, admission systolic blood pressure, left ventricular ejection fraction (LVEF), known kidney dysfunction, baseline hs-CRP, and cardiogenic shock (CS)). The logistic models were assessed for both NGAL concentration as a continued variable (log_2_ transformed to obtain a normal distribution) and for NGAL concentration dichotomized into > versus ≤ the median on admission. Finally, the logistic regression models were reassessed without patients in CS.

The association between the cumulative incidence of KDIGO2-3 and 30-day all-cause mortality as a competing event in patients with admission NGAL plasma concentration > versus ≤ to the median was assessed using Gray’s test for competing risk. The log-rank test was used to compare Kaplan–Meier survival curves between NGAL concentration > and ≤ to the median for the composite endpoint.

The predictive value of admission biomarker plasma concentrations and the composite endpoint were assessed as the area under the receiver operating characteristics curve (AUC_ROC_). The biomarker concentration with the highest combined sensitivity and specificity comprised the optimal concentration for predicting the composite endpoint.

In the subgroup, temporal changes in biomarker plasma concentrations were assessed using a linear mixed model of covariance. The predictive value of NGAL concentration at each time point (baseline, 6–12, and 12–24 h after admission) and composite endpoint was assessed as AUC_ROC_.

A causal mediation analysis was performed to determine the relative impact of baseline NGAL and hs-CRP plasma concentration on the composite endpoint.

Statistical analyses were performed using RStudio version 2022.07.1 (The R Foundation, Vienna, Austria) and SAS Enterprise software version 7.4 (SAS Institute, Cary, NC, USA). A two-sided *p*-value < 0.05 was considered statistically significant.

## 3. Results

The study included 1892 consecutive STEMI patients. Fifteen patients (0.8%) were excluded due to missing creatinine concentrations. In total, 1764 (94%) patients survived 30 days after index admission, while 113 patients (6%) died within 30 days after admission from various causes. Of surviving patients, 1723 (92%) developed KDIGO0-1, and 41 (2.3%) developed KDIGO2-3 (Figure 1).

NGAL concentration was measured in 1624 patients (86%) on admission and in a consecutive subsample at 6–12 h (n = 163) and 12–24 h (n = 222) after admission. NGAL concentrations on admission were not available for the remaining 253 patients (14%) due to early death, early transfer to local hospital, or assay failure.

When dichotomizing patients into NGAL concentration on admission > versus ≤ the median of 79 (56; 118) ng/mL (Table 1), patients with NGAL concentration > the median were older and more frequently found to have hypertension, peripheral artery disease (PAD), IHD, known kidney dysfunction (creatinine levels > 100 mmol/L and/or dialysis treatment), heart failure, and previous stroke. These patients were in worse hemodynamical conditions with lower systolic blood pressure, lower LVEF, and higher heart rate, more likely to have anterior STEMI, multi-vessel disease, and to be comatose after cardiac arrest, and in CS.

The composite endpoint occurred in 107 (13%) patients with NGAL > the median compared with 22 patients (2.7%) with NGAL ≤ the median, *p* < 0.001 (Table 1). The proportion of patients alive 30 days after admission but who developed KDIGO2-3 (n = 36) was similarly higher in patients with NGAL > the median (31 (3.9%) vs. 5 (0.6%), *p* < 0.001).

**Figure 2:**
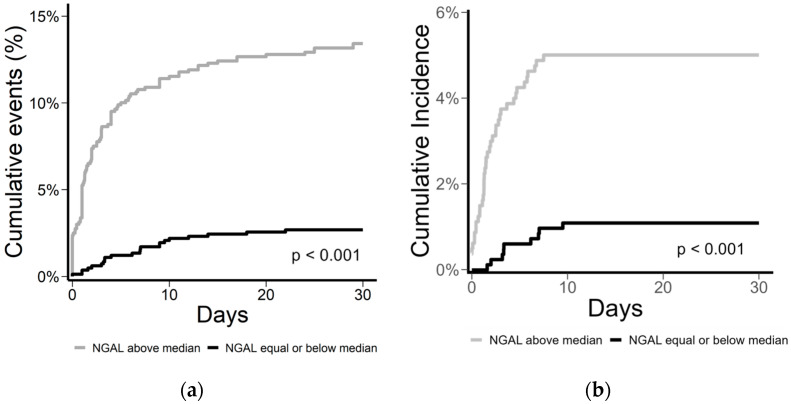
(**a**) Cumulative events (%) of the composite endpoint (AKI (KDIGO2-3) or 30-day all-cause mortality) and (**b**) cumulative incidence (%) of AKI (KDIGO2-3) with 30-day all-cause mortality as competing risks in STEMI-patients with NGAL plasma concentration > versus ≤ the median on admission.

In the logistic regression model, both dichotomized admission NGAL plasma concentration (odds ratio (OR) (95% confidence interval (CI) 5.64 (3.60–9.25), *p* < 0.001 (univariable) and 2.26 (1.18–4.51), *p* = 0.014 (multivariable)) and log_2_ transformed NGAL concentration (3.16 (2.59–3.90), *p* < 0.001 (univariable) and 1.75 (1.24–2.45), *p* = 0.002 (multivariable)) were associated with an increased risk of the composite endpoint (Table 2). The logistic regression models were reassessed excluding patients in CS, with similar results. In a cumulative incidence plot (Figure 2), we found cumulative events and incidence to be associated with patients with NGAL > the median.

Considering the two endpoints (KDIGO2-3 and 30-day mortality) separately, we found both log_2_ transformed and dichotomized NGAL concentrations on admission to be associated with 30-day all-cause mortality in both the univariable and the multivariable model. Regarding KDIGO2-3, both log_2_ transformed and dichotomized NGAL concentrations on admission were associated with KDIGO2-3 in the univariable model but not in the multivariable model: *p* = 0.12 (log_2_ transformed) and *p* = 0.31 (dichotomized) (Table 3).

**Figure 3:**
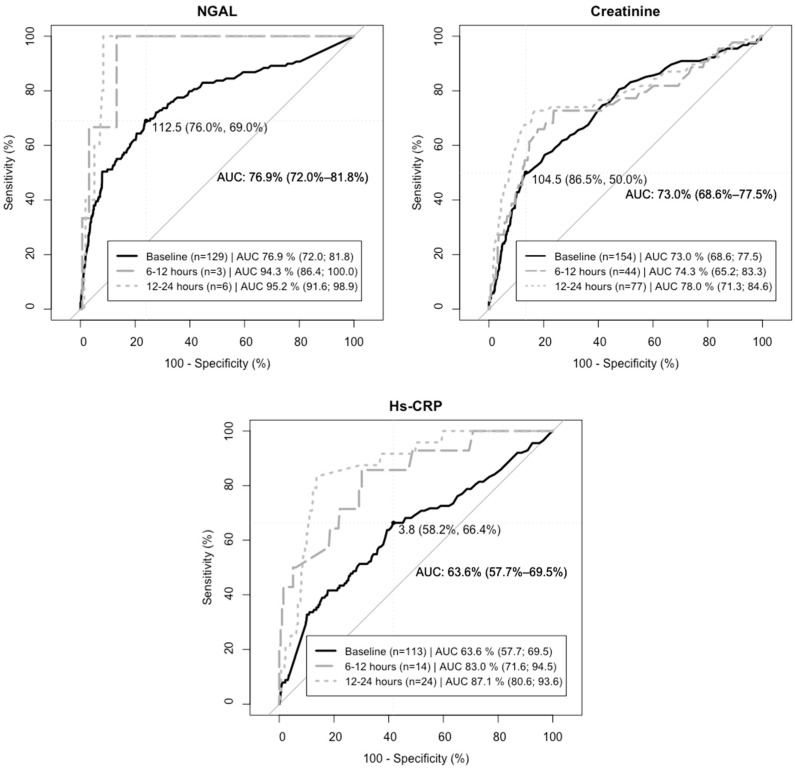
Prediction of composite endpoint (KDIGO2-3 or 30-day all-cause mortality) based on NGAL plasma levels on admission (baseline), 6–12 h, and 12–24 h after admission. The area under the receiver operating characteristic (AUC_ROC_) curve represents the predictive value for NGAL, creatinine, and hs-CRP plasma concentrations measured on admission, 6–12 h, and 12–24 h after admission. The number of patients (n) with composite endpoint and available plasma concentrations at the time points (baseline; 6–12 h, 12–24 h) were NGAL (n = 129; 3; 6); creatinine (n = 154; 44; 77); hs-CRP (n = 113; 14; 24). Dashed lines mark the sensitivity and 100%-specificity for optimal biomarker cut-off concentrations on admission/baseline (NGAL 112.5 ng/mL (specificity 76.0%, sensitivity 69.0%), creatinine 104.5 mg/L (specificity 86.5%, sensitivity 50%), hs-CRP 3.8 µmol/L (specificity 58.2%, sensitivity 66.4%).

NGAL concentration on admission had a predictive value of composite endpoint of (AUC_ROC_) 0.77 (95% CI: 0.72–0.82) with an optimal cut-off concentration (OCOC) of 112.5 ng/mL (specificity, sensitivity) (76.0%, 69.0 %) (Figure 3). Regarding KDIGO2-3 and 30-day all-cause mortality separately, the predictive values of admission NGAL concentration were 0.72 (0.63–0.81) and 0.78 (0.72–0.84), respectively.

**Figure 4:**
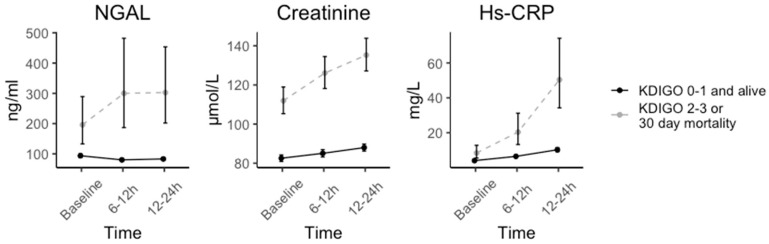
Temporal changes in biomarkers in patients developing KDIGO2-3 or 30-day mortality versus KDIGO0-1. The number of patients (n) with available plasma concentrations and outcome (KDIGO0-1 versus composite endpoint) at time points (baseline; 6–12 h, 12–24 h) were NGAL (n = 1495; 159, 216) vs. (n = 129; 3; 6); creatinine (n = 1723; 312; 828) vs. (n = 154; 44; 77); hs-CRP (n = 1674; 199; 309) vs. (n = 113; 14; 24).

Temporal changes in biomarkers for patients by outcome are depicted in Figure 4. Regarding NGAL, we observed immediately increasing concentrations of NGAL in the subsequent twelve hours for patients with the primary composite endpoint, whereas no increase was observed in patients without a primary composite endpoint event. The predictive values for composite endpoint increased during the first day of hospitalization for all biomarkers. However, the number of cases in the subgroup is low (n = 163; 6–12 h after admission, n = 222; 12–24 h after admission), limiting the interpretation (Figure 3).

**Figure 5:**
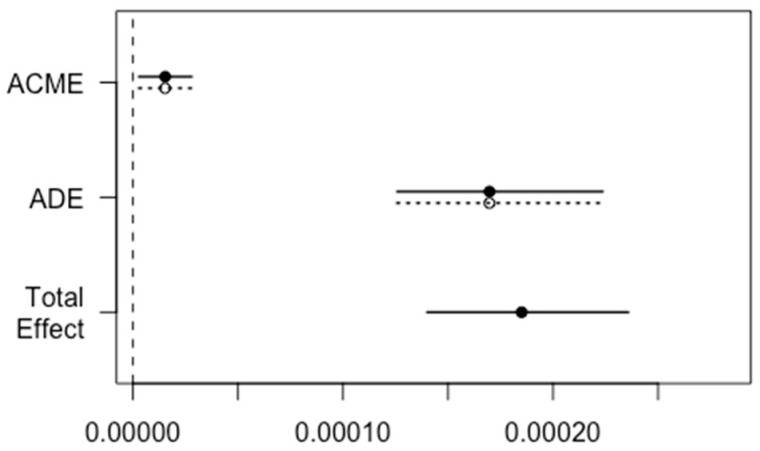
Causal mediation analysis of the relative impact of baseline NGAL and hs-CRP plasma concentration on the composite endpoint (KDIGO2-3 and 30-day all-cause mortality). ACME (average causal mediation effect) is the indirect effect of baseline NGAL plasma concentrations on the composite endpoint (KDIGO2-3 and 30-day all-cause mortality) mediated through hs-CRP on admission. ADE (average direct effect) is the direct effect of baseline NGAL plasma concentrations on the composite endpoint. The dashed line represents the control, and the solid line represents the treated samples.

Through causal mediation analysis, the direct effect of baseline NGAL on the composite endpoint (ADE: average direct effect) was estimated to 0.00017 (95% CI: 0.00012–0.00022) (*p* < 0.001) and the indirect effect of baseline NGAL plasma concentrations through baseline hs-CRP (ACME: average causal mediation effect) was estimated to 0.000015 (0.0000039; 0.000030) (*p* = 0.01), leaving the relative effect of the mediator hs-CRP through NGAL to be 0.088 (0.033; 0.14) (*p* = 0.006). The causal mediation effect is visualized in Figure 5.

## 4. Discussion

In a cohort of more than 1600 consecutive STEMI patients, we found admission NGAL plasma concentrations > the median to be independently associated with a higher risk of severe AKI (KDIGO2-3) and death from all causes within 30 days. Finally, our data indicate the predictive value of NGAL plasma concentration to be higher 6–24 h after index admission.

NGAL is a biomarker released from mature neutrophils during diapedesis and from nephrons during renal injury and ischemia [4,5]. NGAL is accumulated and detected earlier after kidney injury compared with plasma creatinine [5] and can differentiate between structural and functional kidney injuries such as ischemia [16]. In this study, 403 patients had levels of NGAL on admission between 100 and 200 ng/mL, representing a cardiac surgery-associated NGAL (CSA-NGAL) score of 1 and possible tubular damage, while 144 patients had NGAL levels on admission > 200 ng/mL, indicating a CSA-NGAL score of 2–3 and significant or severe tubular damage [17].

Patients with increased concentrations of NGAL (>the median) had higher baseline hs-CRP, which corresponded with previous studies [18,19]. Hs-CRP is known to be associated with AKI and ongoing inflammation. NGAL may reflect a combination of tubular injury and inflammatory responses. To minimize the effect of inflammation on the relationship between NGAL and the composite endpoint (KDIGO2-3 and 30-day all-cause mortality), we included hs-CRP in the multivariable analyses and found NGAL > the median to be independently correlated to the composite endpoint. In the causal mediation analysis, it appears that most of the total effect is explained by the direct effect from baseline NGAL on the composite endpoint; however, there is also a minor indirect effect of hs-CRP on the composite endpoint.

In a study by Cosentino et al., CS was independently correlated with both mortality and AKI [20]. In this study, 9.4% of the total cohort had CS; 3.2% in the group with NGAL ≤ the median and 16% in the group > the median (*p* < 0.001). To eliminate the fact that cardiogenic shock contributes particularly to the development of AKI and death, we adjusted for cardiogenic shock in the multivariable analyses. Furthermore, we assessed the logistic regression analyses excluding patients with CS and found similar OR estimates and *p* values.

In a previous study, 30% of surviving AMI patients developed AKI during hospitalization [2]. In this study, only 10% of surviving STEMI patients developed AKI during hospitalization, perhaps due to fewer comorbidities, younger age, more fortunate geography, and newer techniques of treatment.

In a cohort study of more than 100,000 AMI patients, mild renal dysfunction (defined as glomerular filtration rate (GFR) < 67 mL/min per 1.73 m^2^) increased the 1-year mortality risk by 9%, while severe renal dysfunction (GFR < 31 mL/min per 1.73 m^2^) more than doubled the 1-year mortality risk [21]. Increased NGAL concentrations have previously been predictive of AKI in smaller cohort studies of AMI patients (n < 1050) [6,22,23,24] and increased mortality in STEMI patients [7,8,9,25,26,27]. This study correlates with previous studies of smaller STEMI cohort sizes. To the best of our knowledge, the STEMI population in this study is the largest of its kind.

Since both inflammation and kidney dysfunction are associated with NGAL and unfavorable outcomes in STEMI patients, NGAL plasma concentration may improve the prognostic prediction and identify high-risk patients for whom close follow-ups are essential for survival and nephrotoxic treatment should be considered with great care.

In this study, the predictive values of NGAL plasma concentrations for the composite endpoint trends higher compared with hs-CRP and creatinine both on admission and 6–12 and 12–24 h after admission, which may suggest that NGAL is a better predictor of severe AKI (KDIGO2-3) and 30-day all-cause mortality during index admission. Previously, it has been proposed that NGAL is detected within a few hours of tubular damage before glomerular dysfunction leads to the accumulation of creatinine in the bloodstream [28]. This may explain the lower predictive values of creatinine compared with NGAL. The late accumulation of serum creatinine may cause delayed identification of decreasing GFR, often calculated as Chronic Kidney Disease Epidemiology Collaboration (CKD-EPI) in clinical practice, possibly delaying the initiation of preventive measures of AKI. NGAL has previously been found to be strongly correlated with estimated GFR [29,30].

Additionally, we found increasing predictive values when NGAL concentrations were measured 6–24 h after admission compared with the baseline in a smaller subgroup, suggesting that assessment of NGAL should be delayed for optimal prognostic purposes. However, the size of the subgroup is small, limiting the interpretation; nonetheless, the result invites future studies of the matter.

### Limitations

The primary limitation of this study is the fact that NGAL values at admission were unavailable for 14% of the cohort. Furthermore, we did not adjust for creatinine concentration in the multivariable analyses since we assumed creatinine to be closely correlated to the plasma concentration of NGAL, possibly removing the prognostic signal of NGAL. These limitations may cause an over-or underestimation of the prognostic value in the initial risk stratification. However, our findings match previous results from minor studies.

## 5. Conclusions

NGAL plasma concentration on admission > the median was independently associated with increased risk of severe AKI (KDIGO2-3) and 30-day all-cause mortality in STEMI patients. Furthermore, we observed increasing predictive values of NGAL plasma concentrations in a subgroup during the first day of hospitalization, suggesting that assessment of NGAL should be delayed for optimal prognostic purposes. However, the number of cases in the subgroup were few, limiting the interpretation, and should be verified in future studies.

## Figures and Tables

**Table 1:** Baseline characteristics of STEMI patients stratified into > versus ≤ the median NGAL concentration.

Patient Characteristics	Overall, n = 1624 ^1^	NGAL on Admission (Median = 79 (56; 118) ng/mL)		*p*-Value ^2^
		NGAL ≤ Median, n = 825 ^1^	NGAL > Median, n = 799 ^1^	
Males, n (%)	1203 (74)	607 (74)	596 (75)	0.64
Age (years), mean (SD)	63 (13)	61 (12)	66 (13)	<0.001
Hypertension, n (%)	728 (46)	342 (42)	386 (49)	0.003
Diabetes, n (%)	215 (13)	97 (12)	118 (15)	0.066
Known peripheral artery disease, n (%)	94 (5.9)	33 (4.1)	61 (7.8)	0.002
Previous stroke/TIA, n (%)	114 (7.1)	44 (5.4)	70 (8.9)	0.007
Known kidney disease *, n (%)	75 (4.6)	8 (1.0)	67 (8.4)	<0.001
Known ischemic heart disease, n (%)	232 (14)	103 (12)	129 (16)	0.033
Known heart failure, n (%)	48 (3.0)	17 (2.1)	31 (3.9)	0.030
Multiple vessel disease, n (%)	601 (37)	278 (34)	323 (40)	0.005
Anterior STEMI, n (%)	689 (42)	341 (41)	348 (44)	0.37
Comatose after cardiac arrest, n (%)	89 (5.5)	7 (0.8)	82 (10)	<0.001
CS, n (%)	153 (9.4)	26 (3.2)	127 (16)	<0.001
LVEF (%), median (25; 75)	45 (40, 55)	50 (40, 55)	45 (35, 55)	<0.001
Body Mass Index (kg/m2), median (25; 75)	26.3 (24.1, 29.4)	26.5 (24.2, 29.5)	26.2 (23.9, 29.3)	0.13
Systolic blood pressure on admission (mmHg), median (25; 75)	127 (27)	133 (25)	122 (27)	<0.001
Heart rate on admission (bpm), median (25; 75)	78 (19)	77 (17)	79 (20)	0.015
Time from symptom debut to CAG (min), median (25; 75)	187 (127, 354)	188 (129, 349)	186 (125, 364)	0.77
Plasma NGAL on admission (ng/mL), median (25; 75)	79 (56, 118)	56 (50, 67)	119 (95, 172)	<0.001
Plasma creatinine on admission (mg/L), median (25; 75)	80 (68, 95)	75 (65, 86)	87 (73, 107)	<0.001
Plasma hs-CRP on admission (µmol/L), median (25; 75)	3.1 (1.3, 7.6)	2.5 (1.1, 5.3)	4.2 (1.7, 11.0)	<0.001
KDIGO2-3 or 30-day mortality, n (%)	129 (7.9)	22 (2.7)	107 (13)	<0.001

SD, standard deviation; TIA, transient ischemic attack; LVEF, left ventricular ejection fraction; CS, cardiogenic shock; ^1^ Frequency, n (%); mean (SD); median (25th; 75th percentile); ^2^ Pearson’s Chi-squared test; Wilcoxon rank sum test; * Based on creatinine levels >100 mmol/L and/or dialysis treatment.

**Table 2:** Multivariable logistic regression models for primary composite outcome; AKI (KDIGO2-3) or 30-day all-cause mortality.

Composite Endpoint	OR	95% CI	*p* Value
Log2 transformed NGAL	1.75	1.24–2.45	0.002
Age	1.04	1.02–1.07	<0.001
Systolic blood pressure on admission	1.00	0.99–1.01	0.51
LVEF on admission	0.95	0.93–0.98	<0.001
CS	19.3	10.1–37.7	<0.001
Known kidney disease	1.34	0.47–3.54	0.57
Hs-CRP on admission	1.01	1.00–1.01	0.14
Dichotomized NGAL (>versus ≤ the median)	2.26	1.18–4.51	0.014
Age	1.04	1.02–1.07	<0.001
Systolic blood pressure on admission	1.00	0.99–1.01	0.69
LVEF on admission	0.96	0.94–0.98	<0.001
CS	21.6	11.4–42.1	<0.001
Known kidney disease	1.94	0.72–4.77	0.18
Hs-CRP on admission	1.01	1.00–1.01	0.037

CI: confidence interval; OR: odds ratio. In the univariable model with log_2_ transformed NGAL, we found a doubling of NGAL plasma concentration to increase the risk of the composite endpoint by (OR 95% CI) 3.16 (2.59–3.90), *p* < 0.001. In the univariable model with dichotomized NGAL, the risk of the composite endpoint was increased by (OR 95% CI) 5.64 (3.60–9.25), *p* < 0.001. Analyses excluding patients in CS had similar OR estimates and *p* values.

**Table 3:** Multivariable logistic regression model for incidence of AKI (KDIGO2-3) and 30-day all-cause mortality separately.

AKI (KDIGO2-3)	OR	95% CI	*p* Value
Log_2_ transformed NGAL	1.47	0.91–2.36	0.12
Age	0.98	0.95–1.01	0.15
Systolic blood pressure on admission	1.01	1.00–1.03	0.12
LVEF on admission	0.99	0.96–1.02	0.31
CS	40.0	14.2–120	<0.001
Known kidney disease	5.67	1.61–18.8	0.040
Hs-CRP on admission	0.99	0.94–1.01	0.33
Dichotomized NGAL (> versus ≤ the median)	1.62	0.64–4.33	0.31
Age	0.98	0.95–1.01	0.21
Systolic blood pressure on admission	1.01	1.00–1.03	0.15
LVEF on admission	0.99	0.96–1.02	0.55
CS	44.7	16.1–133	<0.001
Known kidney disease	7.40	2.29–22.5	0.001
Hs-CRP on admission	0.99	0.95–1.01	0.46
**30-day all-cause mortality**	**OR**	**95% CI**	***p*** **Value**
Log_2_ transformed NGAL	1.88	1.29–2.74	0.001
Age	1.07	1.04–1.10	<0.001
Systolic blood pressure on admission	1.00	0.98–1.01	0.62
LVEF on admission	0.95	0.93–0.97	<0.001
CS	10.4	5.03–21.9	<0.001
Known kidney disease	0.51	0.14–1.65	0.28
Hs-CRP on admission	1.01	1.00–1.01	0.091
Dichotomized NGAL (>versus ≤ the median)	2.50	1.14–5.94	0.021
Age	1.07	1.03–1.08	<0.001
Systolic blood pressure on admission	0.99	0.99–1.01	0.40
LVEF on admission	0.95	0.93–0.97	<0.001
CS	11.8	5.98–21.6	<0.001
Known kidney disease	0.82	0.26–2.14	0.73
Hs-CRP on admission	1.01	1.00–1.02	0.018

In the univariable model with log_2_ transformed NGAL, we found a doubling of NGAL plasma concentration to increase the risk of KDIGO2-3 by (OR 95% CI) 2.48 (1.92–3.20), *p* < 0.001, while the risk of 30-day all-cause mortality was increased by (OR 95% CI) 3.12 (2.51–3.91), *p* < 0.001. In the univariable model with dichotomized NGAL, the risk of KDIGO2-3 was increased by (OR 95% CI) 4.78 (2.41–10.6), *p* < 0.001, while the risk of 30-day all-cause mortality was increased by (OR 95% CI) 5.92 (3.48–10.8), *p* < 0.001. Analyses excluding patients in CS had similar OR estimates and *p* values.

## Data Availability

Not applicable.
